# Psychological predictors of delayed active treatment following active surveillance for low‐risk prostate cancer: The Patient REported outcomes for Prostate cARE prospective cohort study

**DOI:** 10.1002/bco2.124

**Published:** 2021-12-14

**Authors:** Kathryn L. Taylor, George Luta, Vasiliki Zotou, Tania Lobo, Richard M. Hoffman, Kimberly M. Davis, Arnold L. Potosky, Tengfei Li, David Aaronson, Stephen K. Van Den Eeden

**Affiliations:** ^1^ Cancer Prevention and Control Program, Lombardi Comprehensive Cancer Center Georgetown University Washington District of Columbia USA; ^2^ Department of Biostatistics, Bioinformatics, and Biomathematics, Lombardi Comprehensive Cancer Center Georgetown University Washington District of Columbia USA; ^3^ Division of General Internal Medicine University of Iowa Carver College of Medicine/Iowa City VA Medical Center Iowa City Iowa USA; ^4^ Department of Urology Kaiser Permanente East Bay Oakland California USA; ^5^ Division of Research Kaiser Permanente Northern California Oakland California USA; ^6^ Department of Urology UCSF San Francisco California USA

**Keywords:** active surveillance, active treatment, anxiety, low‐risk prostate cancer, quality of life

## Abstract

**Objectives:**

In a prospective, comparative effectiveness study, we assessed clinical and psychological factors associated with switching from active surveillance (AS) to active treatment (AT) among low‐risk prostate cancer (PCa) patients.

**Methods:**

Using ultra‐rapid case identification, we conducted pretreatment telephone interviews (*N* = 1139) with low‐risk patients (PSA ≤ 10, Gleason≤6) and follow‐up interviews 6–10 months post‐diagnosis (*N* = 1057). Among men remaining on AS for at least 12 months (*N* = 601), we compared those who continued on AS (*N* = 515) versus men who underwent delayed AT (*N* = 86) between 13 and 24 months, using Cox proportional hazards models.

**Results:**

Delayed AT was predicted by time dependent PSA levels (≥10 vs. <10; HR = 5.6, 95% CI 2.4–13.1) and Gleason scores (≥7 vs. ≤6; adjusted HR = 20.2, 95% CI 12.2–33.4). Further, delayed AT was more likely among men whose urologist initially recommended AT (HR = 2.13, 95% CI 1.07–4.22), for whom tumour removal was very important (HR = 2.18, 95% CI 1.35–3.52), and who reported greater worry about not detecting disease progression early (HR = 1.67, 1.05–2.65). In exploratory analyses, 31% (27/86) switched to AT without evidence of progression, while 4.7% (24/515) remained on AS with evidence of progression.

**Conclusions:**

After adjusting for clinical evidence of disease progression over the first year post‐diagnosis, we found that urologists' initial treatment recommendation and patients' early treatment preferences and concerns about AS each independently predicted undergoing delayed AT during the second year post‐diagnosis. These findings, along with almost one‐half undergoing delayed AT without evidence of progression, suggest the need for greater decision support to remain on AS when it is clinically indicated.

## 1 INTRODUCTION

The use of active surveillance (AS) to manage low risk prostate cancer (PCa) has increased over the past decade, resulting in fewer men receiving surgery or radiation immediately following the diagnosis.^1^, ^2^, ^3^ This change is the result of several factors, including data indicating that active treatments are associated with substantial treatment side effects that can impair quality of life (QOL)^4^, ^5^, ^6^, ^7^ and the observational^8^, ^9^, ^10^, ^11^, ^12^, ^13^ and randomized studies^14^, ^15^ that have not found a mortality benefit of active treatment (AT) over observation for men with localized PCa.^7^, ^16^ However, decisions about selecting and then continuing on AS remain challenging for men with low‐risk PCa, as they must weigh the harms of potentially unnecessary treatment against their anxiety about not actively treating the cancer.^17^, ^18^, ^19^

Formal AS protocols include monitoring the cancer via periodic PSA tests, digital rectal exams, prostate biopsies, and MRI.^4^, ^5^, ^13^, ^20^, ^21^, ^22^, ^23^ Monitoring provides the option to undergo curative treatment and is based on evidence of disease progression and on patient and physician preferences. Several studies have shown that disease progression results in switching from AS to surgery or radiation.^13^, ^20^, ^23^, ^24^ Among the few studies that have conducted a longitudinal assessment of the role of patient preferences and anxiety in switching to AT, there is evidence of discontinuing AS due to anxiety or personal preference and without evidence of disease progression.^23^, ^24^, ^25^ However, not all studies have found strong evidence showing that PCa‐related anxiety results in being more likely to opt out of AS.^26^, ^27^ Similarly, two recent reviews reached differing conclusions regarding the role of anxiety among men who discontinue AS.^18^, ^19^ In a meta‐analysis, Simpkin and colleagues^18^ concluded that an average of 20% of patients discontinue AS due to anxiety or choice in the absence of disease progression. However, Kinsella and colleagues^19^ concluded that fear of progression has not been definitively shown to contribute to discontinuation of AS in the absence of progression.

Based on these differing conclusions, additional investigation of the role that psychological factors may play in delayed treatment decisions is needed. We have addressed several of the limitations that have been present in earlier studies on switching from active surveillance to active treatment. This study included the following strengths: clinical progression measures were included in multivariable models, only low‐risk cases were included (intermediate cases were excluded), a large sample size, and a prospective assessment of psychological variables and reasons for discontinuing AS.

We conducted the Patient REported outcomes for Prostate cARE (PREPARE) study, a prospective, comparative effectiveness study conducted within an integrated health system. The primary objective was to assess decision‐making factors and patient‐reported outcomes among men with low‐risk PCa.^28^, ^29^, ^30^, ^31^ Here we present the demographic, clinical, and psychological predictors of undergoing AT after having been on AS for at least 12 months. We hypothesized that, after accounting for baseline disease characteristics and subsequent disease progression, increased PCa‐related anxiety, physician treatment recommendations, decisional uncertainty, and personal preferences regarding disease‐related dysfunction would predict switching from AS to AT by 24 months. We also conducted exploratory analyses comparing men who switched to AT without a clinical progression versus those who remained on AS in the presence of a clinical progression.

## 2 METHODS

### 2.1 Participants

We enrolled subjects from Kaiser Permanente Northern California (KPNC) from May 2012 to May 2014. Inclusion criteria were (1) a new diagnosis of low‐risk PCa (≤ stage T2a, PSA ≤ 10 ng/ml, Gleason ≤6); (2) ability to provide informed consent; (3) English speaking. Exclusion criteria were (1) already started PCa treatment; (2) diagnosis via transurethral resection of the prostate, with no subsequent biopsy; (3) KPNC membership ending without evidence of treatment (excluded to avoid potential misclassification of patients who were no longer KPNC patients during the study period); and (4) physician refusal (see below). Details of the exclusions and refusals have been presented previously.^29^

### 2.2 Procedures

**FIGURE 1:**
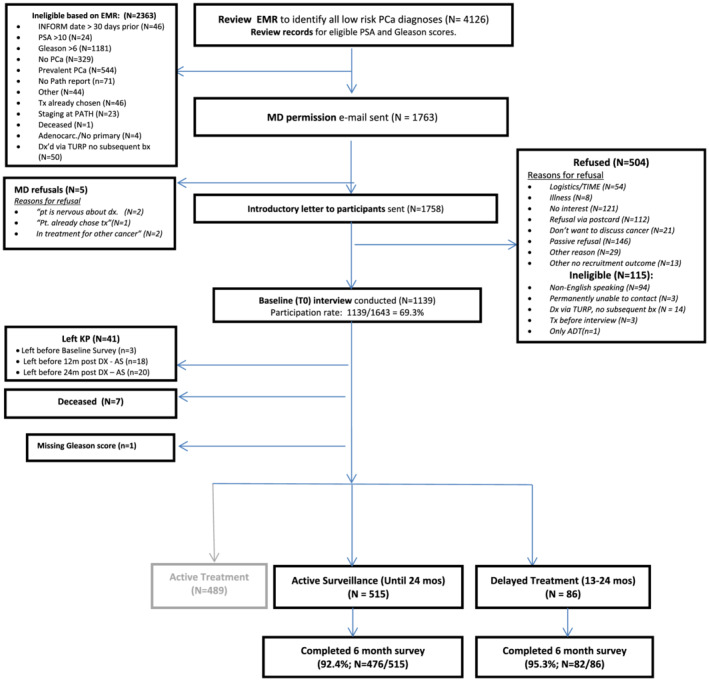
Flow diagram for participants

We used an ultra‐rapid identification process that electronically identified putative cases by twice weekly reviewing pathology data for evidence of prostate biopsies and surgeries (Figure 1). All cases were subsequently linked with the KPNC Cancer Registry to remove prevalent cases and then reviewed to ensure that they met study eligibility criteria. After confirming that patients had been informed of the diagnosis by the treating urologist, we mailed an invitation letter with a return postcard to provide the opportunity to decline participation. We sought to conduct the baseline telephone assessment within 30 days of the patient's notification of his diagnosis, and all were completed prior to treatment. The baseline assessment required 30–40 min and men received a $20 gift card.

The follow‐up assessment was completed 6–10 months post‐diagnosis (*M* = 6.9, SD = 0.8; 6–7 months (68%), 8 months (24%), and 9–10 months (8%)). These assessments were completed by telephone interview (51%) or by patients on a web‐based platform (49%) and they required 20–30 minutes to complete. Participants received a $10 gift card. Participants also completed a 24‐month follow‐up assessment (not presented here). IRB approval was received from the Kaiser Foundation Research Institute. All patients provided informed consent for study participation.

### 2.3 Analytical Cohort (Figure 1)

Of the 1643 eligible men diagnosed with low‐risk PCa from May 2012 to May 2014 at KPNC, we enrolled 1139 (69.3% participation rate) and followed the cohort for 2 years (until May 2016). Exclusions included the <5% who left KPNC or died before 24 months post‐diagnosis while on AS and men who underwent immediate AT (*N* = 489).

## 3 MEASURES

### 3.1 Demographic and clinical characteristics

We elicited demographic characteristics from participants and abstracted EHR‐based clinical information. We used the Elixhauser Comorbidity Index^32^ to calculate a comorbidity score, based on 30 chronic health conditions present in the EHRs, from 1 year pre‐diagnosis to 60 days post‐diagnosis.

### 3.2 Treatment groups and surveillance testing

We abstracted EHR information on PCa treatments and surveillance testing that occurred between diagnosis and 24 months post‐diagnosis. The treatment groups are (1) Continued Active Surveillance for 24 months (Continued AS), defined as the presence of surveillance PSAs and/or biopsies and no active treatment; and (2) AS for a minimum of 12 months (with surveillance PSAs and/or biopsies and no active treatment) followed by PCa treatment(s) between 13 and 24 months post‐diagnosis (Delayed AT).

During the study period, a uniform surveillance protocol for AS was not yet in place across all 21 KPNC medical centers, and thus surveillance procedures were determined by individual clinicians. The diagnostic Gleason score was from the biopsy immediately preceding the PCa diagnosis. The surveillance biopsy that occurred closest to 24 months post‐diagnosis was considered the final Gleason score for the Continued AS group, and the biopsy immediately preceding treatment was the final Gleason score for the Delayed AT group. A Gleason score increase of ≥1 was classified as disease progression. Diagnostic and surveillance biopsies included a minimum of 12 cores.

The baseline PSA (ng/ml) was measured immediately preceding the diagnosis. PSA doubling time (<36 months vs. ≥36 months) was calculated using a minimum of the last two PSAs prior to 24 months post‐diagnosis for the Continued AS group, and prior to treatment for the Delayed AT group.

### 3.3 General psychological outcomes

At both assessments, men completed PROMIS® (Patient‐Reported Outcomes Measurement Information System)^33^, ^34^ custom short‐forms for depression (3 items; alpha = 0.87) and anxiety (3 items; alpha = 0.81). Higher scores (*T* scores, mean = 50, SD = 10) indicate greater depression and greater anxiety.

### 3.4 Prostate‐specific anxiety

At each assessment, participants completed five items from the Cancer Control Subscale of the Health Worry Scale (alpha = 0.77).^35^, ^36^ The response scale for each item was 0–4 (‘not at all’ to ‘very much’). A higher score indicates greater prostate‐related anxiety (range = 0–20). We assessed whether the total score and the individual items were associated with undergoing delayed AT.

### 3.5 Health concerns associated with the treatment decision

At baseline, men indicated the importance (‘very’, ‘somewhat’, or ‘not at all’) of several health concerns influencing their treatment decision, including cancer control (*N* = 7; e.g., wanting the cancer removed), treatment‐related quality of life (*N* = 5; e.g., avoiding problems with sexual function) and treatment burden (*N* = 3; e.g., out‐of‐pocket costs).

### 3.6 Decision making variables

At baseline, we measured decisional certainty with the SURE Test,^37^ a four‐item version of the Decisional Conflict Scale (alpha = 0.71). Response categories were ‘yes’ (1) and ‘no’ (0). Scores ≤ 3 indicate decisional conflict. In addition, we measured PCa‐related knowledge (natural history, treatment side effects, and treatment options)^29^. Response choices were ‘true’, ‘false’ or ‘do not know’, with ‘do not know’ scored as incorrect. Correct items were summed for the total score (higher indicates greater knowledge).

## 4 STATISTICAL ANALYSES

### 4.1 Descriptive analyses

**FIGURE 2:**
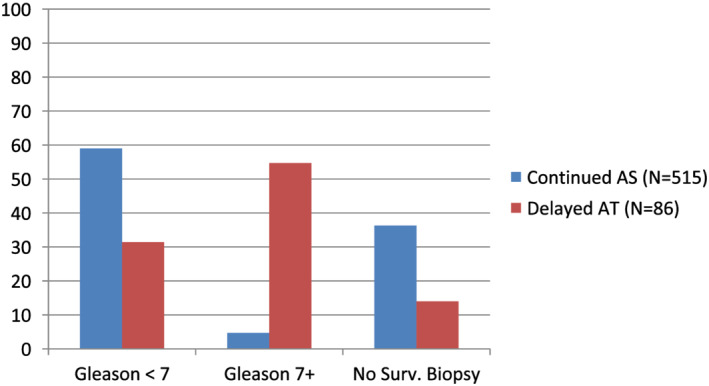
Concordance of surveillance Gleason score with remaining on AS vs. undergoing delayed AT

We compared the two treatment groups (Continued AS vs. Delayed AT) on demographic and clinical characteristics using chi square tests for categorical variables, and t‐tests for continuous variables (Table 1). Table 2 includes the surveillance procedures and results, Table 3 includes descriptive statistics for the psychological variables, and Table S1 includes the health concerns data. We had very little missing data at each assessment (<1% with the exception of income) and high retention at 6 months (Figure 1). Cox proportional hazard models included men who completed both assessments and for whom we had complete EHR data at 24 months post‐diagnosis. Finally, we explored the characteristics of men who switched to AT without a clinical progression and those who remained on AS in the presence of a clinical progression (Figure 2).

**TABLE 1:** Baseline demographic and clinical characteristics

			Treatment group				
	All		Continued active surveillance		Delayed treatment		
	N	col %	N	col %	N	col %	*P* value
All	601	100	515	100	86	100	
Age at diagnosis							0.54
<60 years old	213	35.4	180	35	33	38.4	
60+ years old	388	64.6	335	65	53	61.6	
Race							0.49
Non‐white	114	19	100	19.4	14	16.3
White	487	81	415	80.6	72	83.7	
Hispanic							0.52
No	538	90.3	462	90.6	76	88.4	
Yes	58	9.7	48	9.4	10	11.6	
Marital status							0.84
Married (or living as married)	486	81	417	81.1	69	80.2	
Not married (single, widowed, divorced, separated)	114	19	97	18.9	17	19.8	
Education							0.61
Grad school/degree	164	27.5	145	28.3	19	22.4	
4‐year college degree	134	22.4	115	22.5	19	22.4	
Some college/2 years							
College	183	30.7	156	30.5	27	31.8	
High school or less	116	19.4	96	18.8	20	23.5	
Employment							0.59
Employed	360	60.9	306	60.5	54	63.5	
Not employed	231	39.1	200	39.5	31	36.5	
Income^a^							0.84
$125 001+	157	28.1	137	28.5	20	25.6	
$75 001–$125 000	196	35.1	169	35.2	27	34.6	
≤$75 000	205	36.7	174	36.3	31	39.7	
Elixhauser index^b^							0.88
0	202	33.6	173	33.6	29	33.7	
1	166	27.6	144	28	22	25.6	
2+	233	38.8	198	38.4	35	40.7	
First degree relative with prostate cancer							0.41
Yes	153	25.5	128	24.9	25	29.1	
Prior cancer (not PCa)							0.89
Yes	37	6.2	32	6.2	5	5.8	
Diagnostic PSA level							0.91
8–10	84	14	71	13.8	13	15.1	
6–7	208	34.6	177	34.4	31	36	
4–5	245	40.8	213	41.4	32	37.2	
<4	64	10.6	54	10.5	10	11.6	
Diagnostic Gleason							0.48
6	598	99.5	512	99.4	86	100	
≤5	3	0.5	3	0.6	0	0	
Clinical T‐Stage^7^ at diagnosis							0.21
T1c	561	93.7	484	94.2	77	90.6	
T2a	38	6.3	30	5.8	8	9.4	
Number positive cores–diagnostic biopsy							0.047
3+	151	25.1	122	23.7	29	33.7	
≤2	450	74.9	393	76.3	57	66.3	
Urologist Initial Recommendation (patient self‐report at baseline)							0.039
AS	208	34.6	185	35.9	23	26.7	
AT	83	13.8	63	12.2	20	23.3	
Do not know/	212	35.3	183	35.5	29	33.7	
No recommendation/patient should decide							
No discussion yet	98	16.3	84	16.3	14	16.3	
With urologist							

^a^ *N* = 35 missing in AS group and *N* = 8 missing in Delayed group.

^b^ Comorbid illnesses from 1 year pre‐diagnosis to 60 days post‐diagnosis in EMR.

**TABLE 2:** Surveillance procedures and results

			Treatment group				
	All		Continued active surveillance		Delayed treatment		
	N	%	N	%	N	%	*P* value
All	601	100	515	100	86	100	
*N* of surveillance biopsies							0.0001
0	199	33.1	187	36.3	12	14	
1	361	60.1	290	56.3	71	82.6	
2+	41	6.8	38	7.4	3	3.5	
*N* of surveillance PSA tests							0.013
1–5	123	20.5	99	19%	24	28%	
6–10	386	64.2	329	64%	57	66%	
11–21	92	15.3	87	17%	5	6%	
Final Gleason score^a^							0.0001
<7	331	55	304	59	27	31.4	
≥7	71	12	24	5	47	54.7	
No surveillance biopsy	199	33	187	36	12	14	
Final PSA level							0.0004
<4	178	29.6	164	31.8	14	16.3	
4 to <10	393	65.4	331	64.3	62	72.1	
10+	30	5	20	3.9	10	11.6	
PSA doubling time^b^							0.009
≥36 mos/decrease in PSA	486		425	83	61	71	
≤35 mos	113		88	17	25	29	
Time between diagnosis and treatment							n/a
13–18 months		n/a			52	60.5	
19–24 months		n/a			34	39.5	
Treatment modality							n/a
Radical prostatectomy		n/a			44	51%	
Radiation therapy		n/a			35	41%	
Androgen deprivation		n/a			1	1%	
Combination		n/a			6	7%	
Number of biopsies			0.7	0.6	0.9	0.4	0.006
Number of PSAs			8	2.8	6.9	2.6	0.001

^a^ Last Gleason score, without using baseline diagnostic Gleason score.

^b^ PSA doubling time: 2 years before diagnosis to treatment (DT group) or up to 24 months post‐diagnosis (AS group).

**TABLE 3:** Psychological and decision‐making variables stratified by treatment group

Variable		Continued AS (*N* = 515)	Delayed Treatment (*N* = 86)	*P* value
**Psychological**				
PROMIS anxiety (higher = more anx)	Baseline	51.0 (8.52)	51.2 (8.63)	0.82
	Follow‐up	48.6 (8.18)	49.7 (8.38)	0.27
PROMIS depression (higher = more depr)	Baseline	48.3 (8.6)	48.4 (8.0)	0.86
	Follow‐up	47.1 (7.86)	47.9 (8.33)	0.37
Clark PCa Anxiety total; (hi = more anx.)	Baseline	10.7 (4.4)	11.0 (4.2)	0.57
	Follow‐up	10.5 (4.0)	11.3 (3.9)	0.12
Clark PCa Anxiety individual items				
Worry about dying before my time.	Baseline: Not at all/A little Somewhat/Quite a bit/Very much	67.7% 32.3%	59.3% 40.7%	0.13
	Follow‐up: Not at all/A little Somewhat/Quite a bit/Very much	75.2% 24.8%	68.3% 31.7%	0.19
Worry about what my doctor will find next.	Baseline: Not at all/A little Somewhat/Quite a bit/Very much	61.5% 38.5%	56.9% 43.0%	0.43
	Follow‐up: Not at all/A little Somewhat/Quite a bit/Very much	65.6% 34.5%	63.4% 36.6%	0.71
Worry that changes in medical condition will not be detected early.	Baseline: Not at all/A little Somewhat/Quite a bit/Very much	61.5% 38.5%	52.3% 47.7%	0.11
	Follow‐up: Not at all/A little Somewhat/Quite a bit/Very much	67.4% 32.6%	52.4% 47.6%	0.008
Live in fear that my PSA will rise.	Baseline Not at all/A little Somewhat/Quite a bit/Very much	64.0% 35.9%	63.9% 36.1%	0.99
	Follow‐up: Not at all/A little Somewhat/Quite a bit/Very much	67.9% 32.1%	68.3% 31.7%	0.94
Confident that my cancer can be kept under control.	Baseline: Not at all/A little Somewhat/Quite a bit/Very much	70.9% 29.0%	67.4% 32.6%	0.51
	Follow‐up: Not at all/A little Somewhat/Quite a bit/Very much	56.4% 43.6%	48.8% 51.2%	0.20
**Baseline Health Concern** (see Table S1 for additional Health Concerns)				
Want the cancer removed from your body	Not at all/Somewhat important Very important	253 (49.5%) 258 (50.5%)	31 (36.1%) 55 (63.9%)	0.049

### 4.2 Outcome models

To assess the predictors of treatment group (Continued AS vs. Delayed AT) at 24 months post‐diagnosis, we used two Cox proportional hazard models to estimate adjusted hazard ratios and construct 95% confidence intervals (Table 4). Model 1 included the two time dependent covariates of PSA and Gleason scores, in addition to age, race, the Elixhauser Comorbidity assessment, and men's baseline self‐report of their urologist's treatment recommendation. Model 2 added the two decisional and psychological variables found to have a significant bivariate association with treatment group: wanting the cancer removed, and worry that changes in one's medical condition would not be detected early.

**TABLE 4:** Results from COX proportional hazards models predicting delayed treatment

Variables	Categories	Model 1 DT versus AS (ref)		Model 2 DT versus AS (ref)	
		HR	95% CI	HR	95% CI
**Gleason score** (time dependent) (ref = <7)	7+	**20.18**	**12.60, 32.33**	**23.86**	**14.27, 39.89**
	No surveillance biopsy	0.93	0.47, 1.86	0.57	0.27, 1.22
**PSA level** (time dependent) (ref = <4)	4 to 10	1.52	0.84, 2.75	1.58	0.86, 2.93
	10+	**5.33**	**2.34, 12.13**	**5.11**	**2.14, 12.2**
**Age**	Continuous	1.01	0.98, 1.05	1.01	0.97, 1.05
**Race** (ref = non‐white)	White	**2.83**	**1.44, 5.57**	**3.03**	**1.57, 5.85**
**Comorbidities at diagnosis** (ref = 0)	1	1.40	0.79, 2.48	1.43	0.79, 2.58
	2+	1.10	0.67, 1.82	0.95	0.55, 1.64
**Pt reported urologist treatment recommendation at baseline** (ref = AS)*	AT	**1.99**	**1.06, 3.71**	**2.13**	**1.07, 4.22**
**Want the cancer removed** (baseline) (ref = not at all/somewhat)	Very important			**2.18**	**1.35, 3.52**
**Worry changes in medical condition will not be detected early** (6 months) (ref = not at all/ a little)	Somewhat/ Quite a Bit/ Very Much			**1.67**	**1.05, 2.65**

### 4.3 Power calculations

With the sample size of 515 (Continued AS) and 86 (Delayed AT) and using categorical measures of the psychological and decisional predictors, after adjusting for demographic and clinical variables, at a significance level of 0.05, we have 80% power to detect HRs of 1.4 (or 0.7 for inverse associations) for the Continued AS versus Delayed AT comparison. SAS version 9.3 was used for all analyses.

## 5 RESULTS

### 5.1 Participation rates

Of 1644 eligible men, 1139 (69.3%) completed the baseline assessment (Figure 1; median = 24 days post‐diagnosis). Compared to those who declined or could not be reached, participants were more likely to be white (*p* < 0.0001), with no other significant demographic or clinical differences. Detailed information on accrual and retention has been presented previously.^29^, ^30^

### 5.2 Descriptive results

Between 13 and 24 months post‐diagnosis, 14.3% (86/601) underwent delayed treatment and 85.7% (515/601) remained on AS. There were no baseline demographic differences between the groups (Table 1). Regarding clinical characteristics, the Continued AS group had significantly fewer positive cores (*p* < 0.05) and were more likely to report receiving a urologist's recommendation for AS versus AT (p < 0.05). The surveillance procedures (Table 2) during the 24‐month follow‐up period show that the Continued AS group received significantly more PSA tests (*p* = 0.013), while the Delayed AT group was more likely to undergo a surveillance biopsy (*p* < 0.0001) and to have surveillance results that were more suggestive of cancer progression (Table 2).

Regarding the psychological variables, there were no significant group differences at baseline or follow‐up on the total scores of the PROMIS anxiety and depression scales or on the prostate‐specific anxiety scale (Table 3). We also evaluated each of the prostate‐specific anxiety items, observing that at follow‐up those with greater worry about changes in their medical condition not being detected early were significantly more likely to switch to AT, compared to those with less worry (*p* = 0.008).

Decision‐making variables, including baseline health concerns (cancer control, treatment‐related quality of life, and treatment burdens), indicated that men who reported greater importance of ‘wanting the cancer removed from my body’ were more likely to undergo delayed AT (p < 0.05; Table 3). The other health concern items did not predict delayed AT (Table S1). Decisional certainty, PCa knowledge, and baseline treatment preference were not significantly associated with treatment group (Table S2). Finally, prostate‐related QOL measured at baseline or follow‐up^38^ was not associated with switching to AT (data not shown).

### 5.3 Cox Proportional Hazards Models

The Cox models assessed the likelihood of undergoing delayed AT after 12 months of AS (Table 4). In Model 1, the time dependent covariates indicate that a surveillance biopsy (Gleason ≥7) or a PSA (>10) were each independent predictors of delayed AT, as well as white race and reporting having received a urologist's treatment recommendation at diagnosis for AT. Age and the number of comorbid conditions were unrelated to switching.

In Model 2, after adjusting for Model 1 variables, men who rated having the cancer removed as ‘very important’ at baseline were more than twice as likely to undergo AT, compared to those whose rating was ‘somewhat/not at all important’ (HR = 2.18, 95% CI 1.35–3.52). Men with greater ‘worry that changes in my medical condition would not be detected early’ were more likely to undergo delayed AT, compared to those with less worry (HR = 1.67, 95% CI 1.05–2.65).

### 5.4 Exploratory analyses (Figure 2)

We explored whether the decision to remain on AS vs. undergo delayed AT was concordant with surveillance biopsy results (Figure 2). First, among those who switched to AT, 55% (47/86) had evidence of biopsy‐related progression (surveillance biopsy ≥7), while 31% (27/86) had a stable surveillance biopsy (Gleason ≤6), and 14% (12/86) did not have a surveillance biopsy. Thus, 45% (39/86) switched to AT without evidence of progression from a biopsy.

Second, among the men who remained on AS, 59% (304/515) had no evidence of biopsy‐related progression (surveillance biopsy ≤7), 4.7% (24/515) had a surveillance biopsy of 7+, and 36.3% (187/515) did not have a surveillance biopsy (Figure 2). Thus, 4.7% (24/515) remained on AS while having evidence of progression from a biopsy and 36.3% (187/515) remained on AS without having had a surveillance biopsy.

## 6 DISCUSSION

In this prospective cohort study of men undergoing AS for low‐risk PCa, 14% underwent delayed AT by 24 months post‐diagnosis, which is similar to other AS cohorts.^13^, ^23^, ^24^ Although it is well‐documented that disease progression of low‐risk PCa predicts switching from AS to AT,^13^, ^20^, ^23^, ^24^ this is one of few longitudinal studies investigating the role that decisional and psychological factors may play in this decision while accounting for evidence of disease progression.^23^, ^25^, ^26^, ^27^ In multivariable analyses, adjusting for disease progression and urologists' initial treatment recommendation, men's baseline desire to have their cancer removed and their subsequent worry that disease progression would not be detected early each independently predicted undergoing delayed AT during the second year post‐diagnosis. Importantly, there were no differences on general anxiety or depression or overall prostate‐related anxiety between those who continued AS vs. underwent delayed AT, indicating that delaying AT was not associated with greater distress while undergoing AS.

These findings support prior longitudinal studies that have found that fear of disease progression was associated with undergoing delayed AT when the disease had not progressed.^23^, ^25^ In a meta‐analysis that included 26 AS cohorts, Simpkin^18^ concluded that 20% of men discontinue AS due to anxiety. However, not all studies have found that switching to AT was associated with fear of disease progression.^26^, ^27^ The conflicting findings may be associated with analytic differences, including the adjustment for clinical progression in multivariable models versus limiting the analysis to men whose disease had not progressed. Our findings indicate the clinical importance of understanding men's specific prostate‐related anxieties associated with the initial choice to undergo AS, given the potential for its subsequent impact on the decision to discontinue AS in the absence of disease progression. More research is needed to understand the role of psychological factors in discontinuing AS among men with low‐risk PCa, as most of the work on treatment decisions for low‐risk PCa has addressed the initial treatment decision. As an example, the desire to remove the cancer has been associated with selecting AT as the initial treatment,^39^ but we are unaware of studies that have included this variable when assessing delayed AT.

Among men in the Delayed AT group, despite the greater likelihood of having received an initial recommendation for AT and of having an initial preference for the cancer to be removed, they nonetheless remained on AS for a minimum of 12 months. Importantly, the Delayed AT group did not report greater general anxiety or depression compared to the Continued AS group at either the baseline or six‐month follow‐up assessment. These results provide important data for clinicians when discussing the treatment decision with men who are considering AS. Among men who ultimately switch to AT, the likelihood of experiencing increased anxiety or depression during the AS period is low. Providing education about the fact that switching to AT is an option, with or without disease progression, may help men feel comfortable when considering AS as a management option.

These findings confirm the importance of the urologist's recommendation on the treatment decision.^2^, ^40^, ^41^ What is notable is that the recommendation, as reported by the patient, continued to have a significant impact on the treatment decision 1–2 years later, after adjusting for disease progression and PCa‐related concerns. Although we did not measure men's perception of their urologists' subsequent recommendations, this finding provides new information on the long‐term treatment implications of the urologist's initial recommendation, which may not include the patient's treatment preferences.^40^

In exploratory analyses assessing whether treatment decisions were concordant with surveillance results, we found that 31% of men who underwent delayed AT did so without clinical evidence of progression, and that 16% of men who had evidence of disease progression continued on AS. During the study period, MRI was not used for surveillance of low risk PCa, and thus was unlikely to have influenced treatment decisions. Of the men who remained on AS but who had not had a surveillance biopsy, the majority had a PSA doubling time indicative of less aggressive cancer, suggesting that a minority of men and their physicians made individualized decisions about delayed treatment that relied on surveillance PSA results and possibly patient preferences. Unfortunately, the number of those making discordant decisions was too small to determine whether psychological factors may have played a role in these decisions.^18^ In order to better understand the role of PCa‐related anxiety in switching from AS to AT, we suggest that an important analysis is the comparison of PCa‐related anxiety among four groups: disease progression (yes vs. no) by treatment decision (continued AS vs. delayed AT). To our knowledge, this analysis has not been conducted, but will be useful to understand whether fear of disease progression is contributing to discontinuing AS in favour of AT, in the absence of disease progression.^19^

Study limitations include an underrepresentation of non‐white participants, which led to the small number of non‐white participants who switched to AT (*N* = 14) and the need to combine African Americans with other non‐white participants. The unanticipated finding that non‐white men were less likely to undergo delayed treatment is difficult to interpret in light of their lower participation rate and having to collapse different groups. Second, although participants and those who declined did not differ on other demographic or clinical characteristics, men's reasons for declining participation were unknown in almost a quarter of those eligible. Thus, whether the sample may have underrepresented or overrepresented certain characteristics (e.g., anxiety about disease progression) is unknown. Third, although our 24‐month follow‐up period captured only a portion of the men who may have ultimately undergone delayed treatment, the first 2 years post‐diagnosis is an important timeframe in which to assess continued AS vs. delayed AT. This is particularly true given that approximately one‐third of men made treatment decisions that were discordant with the results of their surveillance results. Fourth, this cohort was accrued prior to use of genetic data in making treatment decisions and MRIs used for surveillance, which may result in different treatment decisions than reported here. Fifth, when evaluating the primary hypotheses, we have not adjusted for multiple comparisons. Finally, we did not assess whether patients' and urologists' individual concerns about remaining on AS may have impacted the surveillance procedures (i.e., PSA tests and biopsies) that were utilized.

Methodological strengths include that this is one of the largest prospective samples of low risk PCa patients on AS who were followed and assessed for early predictors of switching to AT. Participants were assessed shortly post‐diagnosis and followed for 2 years, adjusting for time dependent measures of disease progression. Further, few prospective, longitudinal cohorts of men with low‐risk PCa have included decisional and psychological characteristics that may be relevant to the decision to discontinue AS. Finally, conducting this study within the KPNC integrated healthcare system provided ultra‐rapid case ascertainment and data on treatment decisions via the extensive real‐time EHR surveillance. Further, we are better able to isolate the effects of decisional processes and psychological variables on treatment decisions given that KPNC clinicians are salaried providers. Further, as KPNC providers are salaried, the impact of financial incentives on treatment decisions is limited. These strengths outweigh concerns regarding the generalizability of samples drawn from integrated healthcare systems, particularly given that these systems represent an increasingly large proportion of US healthcare settings.^42^

These results have important clinical implications. Continued decision support may be needed for men to remain on AS when it is clinically indicated during the first 2 years of being on AS. Fear of disease progression and wanting the cancer removed independently predicted undergoing delayed treatment after adjusting for clinical progression and urologists' initial recommendation. This suggests the need to support men's decisions through increased physician engagement and providing resources to increase men's comfort with and understanding of the clinical reasoning and data in support of AS. Further, additional physician education on effective communication about AS and predictors of disease progression may be useful. For some men, in lieu of delayed AT, a more aggressive AS regimen or newer tools (e.g., MRI and/or genetic testing among those with a significant family history of prostate, breast, or ovarian cancer), may assist physicians with risk stratification. Finally, a method is needed to assist clinicians in identifying men who may benefit from additional resources to remain on AS when it is clinically indicated.

## CONFLICT OF INTEREST

This manuscript was supported by NIH R01 CA155578‐01 (Multiple PIs: Kathryn L. Taylor, PhD and Stephen K. Van Den Eeden, PhD). There are no other conflicts of interest to report.

## FUNDING INFORMATION

NIH R01 CA155578‐01 (Multiple PIs: Kathryn L. Taylor, PhD and Stephen K. Van Den Eeden, Ph.D.); P30‐CA051008, Survey, Recruitment, and Biospecimen Collection Shared Resource and Biostatistics and Bioinformatics Shared Resource of the Georgetown Lombardi Comprehensive Cancer Center.

## AUTHOR CONTRIBUTIONS

**Kathryn Taylor:** Conceptualization, methodology, writing‐original draft, review and editing, funding acquisition, and supervision; **George Luta:** Methodology and writing‐review & editing; **Vasiliki Zotou:** Data curation and formal analysis; **Tania Lobo:** Data curation and formal analysis; **Richard M. Hoffman:** Methodology, writing‐review & editing, and conceptualization; **Kimberly M. Davis:** Writing‐review & editing and conceptualization; **Arnold L. Potosky:** Methodology, writing‐review & editing, and conceptualization; **Tengfei Li:** Validation and formal analysis; **David Aaronson:** Writing‐review & editing and conceptualization; **Stephen K. Van Den Eeden:** Conceptualization, methodology, funding acquisition, writing‐review & editing, and supervision.

## Supporting information


**Supplemental Table 1:** Baseline Health Concerns Associated with Treatment Decision
**Supplemental Table 2:** Decision Making Variables
