# Supplementary material for: Psychological predictors of delayed active treatment following active surveillance for low‐risk prostate cancer: The Patient REported outcomes for Prostate cARE prospective cohort study
Source: BJUI Compass. 2021 Dec 14;3(3):226–37. doi: 10.1002/bco2.124 (PMC9045562; doi:10.1002/bco2.124)
Supplement: Supplementary file 1 — Supplemental Table 1: Baseline Health Concerns Associated with Treatment Decision Supplemental Table 2: Decision Making Variables [file BCO2-3-226-s001.docx]

| **Supplemental Table 1: Baseline Health Concerns Associated with Treatment Decision** | | | | |
| --- | --- | --- | --- | --- |
| **Variable** | **Importance Rating** | **Continued AS (N=515)** | **Delayed**  **Treatment (N=86)** | P value |
| **Treatment-related quality of life** | | **N %** | **N %** |  |
| Avoiding possible problems with sexual function/intimacy | Not at all/ Somewhat  Very l | 203 39  311 60 | 35 41  51 59 | 0.89 |
| Avoiding possible problems with bowel function | Not at all/ Somewhat  Very l | 127 25  387 75 | 29 34  57 66 | 0.19 |
| Avoiding possible problems with urinary function | Not at all/ Somewhat  Very l | 98 19  415 81 | 82 18  86 14 | 0.44 |
| Avoiding major surgery (pain, complications, infections, anesthesia) | Not at all/ Somewhat  Very l | 269 52  244 47 | 51 59  35 41 | 0.42 |
| Avoiding radiation exposure | Not at all/ Somewhat  Very | 308 60  206 40 | 50 58  36 42 | 0.88 |
| **Cancer Control** | | | | |
| Wanting the cancer removed from your body?* | Not at all/ Somewhat  Very l | 253 49  258 50 | 31 36  55 64 | 0.049 |
| Feel that you are doing something active to treat the PCa | Not at all/ Somewhat  Very l | 241 47  273 53 | 39 45  47 55 | 0.88 |
| Worry that you might regret your treatment/ management decision | Not at all/ Somewhat  Very | 381 74  133 26 | 58 67  28 33 | 0.39 |
| Doing everything you can to increase the quality of your life | Not at all/ Somewhat  Very | 62 12  452 88 | 12 14  74 86 | 0.81 |
| Doing everything you can to increase the length of your life | Not at all/ Somewhat  Very | 124 24  390 76 | 25 29  61 71 | 0.57 |
| Wanting to be cured of the cancer | Not at all/ Somewhat  Very | 136 26  378 73 | 22 26  64 74 | 0.91 |
| Side effects had by friends or family treated for PCa | Not at all/ Somewhat  Very | 395 77  119 23 | 66 77  20 23 | 0.92 |
| **Treatment Burden** | | | | |
| Inconvenience or burden to partner/ family during treatment/recovery | Not at all/ Somewhat  Very | 328 64  186 36 | 53 62  33 38 | 0.85 |
| Length of time to complete treatment | Not at all/ Somewhat  Very | 348 68  165 32 | 54 63  32 37 | 0.55 |
| Out of pocket costs | Not at all/ Somewhat  Very | 389 76  124 24 | 64 74  22 26 | 0.81 |
| *This item is also included in Table 3 and Table 4. | | | | |

| **Supplemental Table 2: Decision Making Variables** | | | | |
| --- | --- | --- | --- | --- |
| **Variable** |  | **Continued AS (N=515)** | **Delayed**  **AT (N=86)** | P value |
| Baseline Decisional Certainty (SURE)  (hi=more certainty) | Mean (SD) | 3.09 (1.24) | 2.97 (1.31) | 0.40 |
| Baseline PCa Knowledge  (hi=more knowledge) | Mean (SD)B | 3.5 (0.83) | 3.5 (0.78) | 0.88 |
| Patient Treatment Preference (baseline) | Active treatment  Active surveillance  DK/Not sure | 37 (7.2%)  340 (66%)  138 (26.8%) | 9 (10.5%)  48 (55.8%)  29 (33.7%) | 0.17 |
